# Statin uses in adults with non-dialysis advanced chronic kidney disease: Focus on clinical outcomes of infectious and cardiovascular diseases

**DOI:** 10.3389/fphar.2022.996237

**Published:** 2022-09-30

**Authors:** Ching-Chung Hsiao, Jih-Kai Yeh, Yan-Rong Li, Wei-Chiao Sun, Pei-Yi Fan, Chieh-Li Yen, Jung-Sheng Chen, Chihung Lin, Kuan-Hsing Chen

**Affiliations:** ^1^ Department of Nephrology, New Taipei Municipal TuCheng Hospital, New Taipei City, Taiwan; ^2^ College of Medicine, Chang Gung University, Taoyuan, Taiwan; ^3^ Division of Cardiology, Department of Internal Medicine, Linkou Chang Gung Memorial Hospital, Taoyuan, Taiwan; ^4^ Division of Endocrinology and Metabolism, Department of Internal Medicine, Linkou Chang Gung Memorial Hospital, Taoyuan, Taiwan; ^5^ Kidney Research Center and Department of Nephrology, Chang Gung Memorial Hospital, Linkou Branch, Taoyuan, Taiwan; ^6^ Center for Artificial Intelligence in Medicine, Chang Gung Memorial Hospital at Linkou, Taoyuan, Taiwan

**Keywords:** statin, non-dialysis chronic kidney disease, infection, infection-related mortality, all-cause mortality

## Abstract

**Background:** Statins are commonly used for cardiovascular disease (CVD) prevention. Observational studies reported the effects on sepsis prevention and mortality improvement. Patients with chronic kidney disease (CKD) are at high risk for CVD and infectious diseases. Limited information is available for statin use in patients with non-dialysis CKD stage V.

**Method:** The retrospective observational study included patients with non-dialysis CKD stage V, with either *de novo* statin use or none. Patients who were prior statin users and had prior cardiovascular events were excluded. The key outcomes were infection-related hospitalization, major adverse cardiovascular events (MACE) (non-fatal myocardial infarction, hospitalization for heart failure, or non-fatal stroke), and all-cause mortality. The data were retrieved from the Chang Gung Research Database (CGRD) from January 2001 to December 2019. Analyses were conducted with Cox proportional hazard regression models in the propensity score matching (PSM) cohort.

**Result:** A total of 20,352 patients with CKD stage V were included (1,431 patients were defined as *de novo* statin users). After PSM, 1,318 statin users were compared with 1,318 statin non-users. The infection-related hospitalization (IRH) rate was 79.3 versus 94.3 per 1,000 person-years in statin users and statin non-users, respectively [hazard ratio (HR) 0.83, 95% confidence interval (CI) 0.74–0.93, *p* = 0.002]. The incidence of MACE was 38.9 versus 55.9 per 1,000 person-years in statin users and non-users, respectively (HR, 0.72; 95% CI 0.62–0.83, *p* < 0.001). The all-cause mortality did not differ between statin users and non-users, but statin users had lower infection-related mortality than non-users (HR, 0.59; 95% CI 0.38–0.92, *p* = 0.019).

**Conclusion:** *De novo* use of statin in patients with non-dialysis CKD stage V reduced the incidence of cardiovascular events, hospitalization, and mortality for infectious disease. The study results reinforced the benefits of statin in a wide range of patients with renal impairment before maintenance dialysis.

## Background

Chronic kidney disease (CKD) is associated with increased risk of death, cardiovascular (CV) events, and hospitalization (Go et al., 2004). Cardiovascular disease (CVD) is the leading cause of death and hospitalization of patients with CKD (Saran et al., 2017). CV mortality accounts for 40%–50% of deaths in patients with advanced CKD (stage V not receiving dialysis) compared with 26% individuals with normal kidney functions (Foley et al., 1998; Thompson et al., 2015; Jankowski et al., 2021).

Statins, hydroxymethylglutaryl coenzyme A (HMG CoA) reductase inhibitors, have proven to extend life and reduce the atherosclerotic CV events in general population. Among patients with CKD, a meta-analysis showed statin reduced CV events by 24% and mortality by 22% (Palmer et al., 2012). However, randomized control trials failed to show benefits of statin treatment in patients undergoing maintenance hemodialysis (Wanner et al., 2005a; Fellstrom et al., 2009; Baigent et al., 2011a). The contradictory results of statin effects in patients with dialysis-dependent CKD or not raise the concern of decreasing statin effectiveness with progression of CKD.

Furthermore, infection is second leading cause of death and morbidity in patients with CKD. An independent graded relationship is observed between a reduced estimated glomerular filtration rate (GFR) and the risks of death or hospitalization for infectious diseases (Dalrymple et al., 2012; Ishigami et al., 2017; Saran et al., 2017). Statin’s pleiotropic effects of anti-inflammatory and anti-microbial properties in experiments may protect patients from severe infection or sepsis. In observational studies, statin use associated with a reduction in the risk of hospitalization for sepsis or pneumonia in patients with CKD (Schurr et al., 2016).

Limited evidence of protective effects from CV and infection diseases is available in advanced CKD patients with *de novo* statin administration. Thus, the study aimed to evaluate the association of *de novo* uses of statin with risks of infection, major CV events, and mortality in patients with advanced CKD (stage V) before maintenance dialysis.

## Materials and methods

### Data source

We conducted a retrospective cohort study with longitudinal data from the Chang Gung Research Database (CGRD). Patient data were obtained from the largest healthcare provider in Taiwan, Chang Gung Memorial Hospital System, comprising three major teaching hospitals and four tertiary-care medical centers, with a total of 10,050 beds and approximately 280,000 admissions per year (Shao et al., 2019). The requirement to obtain informed consent was waived because data in the CGRD that could identify specific patients are scrambled and encrypted before being released to researchers. The study protocol was approved by the Institutional Review Board, Chang Gung Medical Foundation, Taiwan (IRB No. 202101556B0).

### Patient selection and study design

**FIGURE 1:**
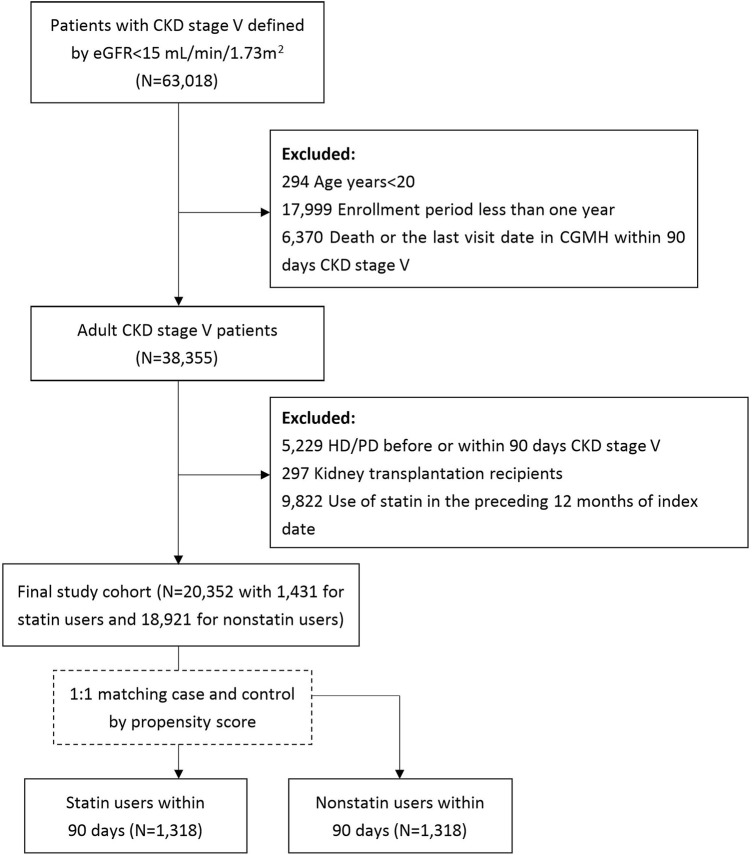
Selection process of study cohort.

Figure 1 demonstrates the process used for selecting the participants in the study cohorts. By searching electronic medical records from the Chang Gung Research Database (CGRD) between 1 January 2001 and 31 December 2019, we retrieved patients with the diagnosis of CKD. The diagnosis of CKD stage V was defined as patients with CKD diagnosis and estimated GFR less than 15 ml/min/1.73 m (Saran et al., 2017) using the CKD-EPI equation (Levey and Stevens, 2010). The index date was defined when patients were diagnosed with CKD stage V. We excluded patients’ age <20 (N = 294), enrollment period less than 1 year (N = 17,999), and death within 90 days of CKD stage V (N = 6,370). Dialysis and kidney transplantation change the risk of infection and major CV events in patients with CKD stage V. Thus, we exclude patients receiving hemodialysis or peritoneal dialysis within 90 days of CKD stage V (N = 5,229) and kidney transplant recipients (N = 297). Patients who used statin in the preceding 12 months of index date (N = 9,822) and patients who had history of CV events (N = 2,655) were also excluded. Finally, 20,352 patients with CKD stage V were enrolled in the study cohort. Statin exposure was defined as CKD V patients who had received the first prescription for statin within 90 days of index date. Among 20,352 patients, 1,431 patients were statin users while 18,921 were statin non-users.

### Covariates and study outcomes

Diseases were detected using International Classification of Diseases, Ninth Revision, Clinical Modification (ICD-9-CM) codes before 2016 or ICD-10-CM codes thereafter. Covariates included age, sex, Charlson Comorbidity Index (CCI), comorbidity, medications, laboratory values, and follow-up years. Comorbidities were identified when reported for more than two outpatient visits or one inpatient stay within the previous year of index date. Most diagnostic codes used for these comorbidities have been validated in previous national database studies (Wu et al., 2014; Hsieh et al., 2015). Medications were identified by the filling of a prescription at least twice or refilling a prescription for a chronic illness at least once 2 years before the index date.

The outcomes of primary interest were infection-related hospitalization (IRH), MACE which were identified based on the principal diagnosis of hospitalization, emergency room visit, and all-cause mortality. MACE was defined by the three components including non-fatal myocardial infarction, hospitalization for heart failure, and non-fatal stroke. The secondary outcomes were CV related mortality and infection-related mortality. CV related mortality and infection-related mortality are ascertained by the main diagnosis in the discharge records for inpatient hospital deaths. All-cause mortality was linked to national death registry. All participants were followed from the index date until the first occurrence of IRH, MACE, death, or the end of the follow-up period (31 December 2019), whichever came first.

### Statistical analysis

Propensity score matching (PSM) was used to reduce the selection bias due to the baseline differences between the statin users and statin non-users. The patient characteristics, comorbidities, medications, and laboratory values between the statin users and non-users groups were compared using the absolute value of standardized mean differences before and after propensity scores matching. The values of standardized differences less than 0.2 were indicating adequate balance between groups. Cumulative probability of IRH during observation period was drawn by statin exposure status. Hazard ratios (HRs) and 95% CIs of major clinical outcomes were compared between groups which used stratified Cox proportional hazard regression models using the matched sets as strata to account for the matching. Since there is a high risk of death in advanced CKD patients, we performed competing risk analysis using the Fine and Gray sub-distributional hazards model to estimate the outcome of IRH, MACE, CV related mortality, and infection-related mortality. Non-CV related mortality was considered a competing event for CV related mortality and non-infection-related mortality was considered a competing event for infection-related mortality. In subgroup analyses, the estimation for risk of IRH and MACE were performed by age, gender, sex, CCI, comorbidities, and statin intensity. The high-statin intensity was defined as patients prescribed of 40–80 mg for atorvastatin or 20–40 mg for rosuvastatin, otherwise was defined as low to moderate intensity. All significance tests were 2-sided, and results were deemed statistically significant if the 95% CI did not overlap 1. The analyses were performed using the SAS Version 9.4 (SAS Institute, Cary, NC).

## Results

### Subject characteristics

Table 1 presents the baseline characteristics of the statin users when compared with the statin non-users. Before PSM, 20,352 patients with CKD stage V met the inclusion criteria and were categorized by their statin exposure status into statin user (*n* = 1,431), and statin non-user (*n* = 18,921) groups. The mean age (years) was 64.1 ± 13.1 in the statin users and 66 ± 14.6 in the statin non-users, respectively. In general, the statin users had higher proportion of CV comorbidities including diabetes mellitus, hypertension, stroke, heart failure, coronary artery disease, myocardial infarction, stroke, and peripheral artery disease. The CCI score was also higher in statin users. Statin users are more likely to receive medications including aspirin/clopidogrel, angiotensin-converting enzyme inhibitors (ACEI)/angiotensin receptor blockers (ARB), beta-blocker, calcium channel blocker, and glucose lowering agents including insulin than statin non-users. After PSM, 1,318 statin users were compared with 1,318 statin non-users as controls. Age, gender proportion, CCI, duration of follow-up, comorbidities, major medications, and laboratory data were similar between the two groups.

**TABLE 1:** Demographic characteristics of study population before and after propensity score matching.

	Before PS matching			After PS matching		
	Statin users (*n* = 1,431)	Statin non-users (*n* = 18,921)	ASMD	Statin users (*n* = 1,318)	Statin non-users (*n* = 1,318)	ASMD
Male, n (%)	684 (47.8)	9,460 (50.0)	0.04	636 (48.3)	626 (47.5)	0.02
Age years, mean ± SD	64.1 ± 13.1	66±14.6	0.14	64.2 ± 13.2	64.3 ± 13.5	0.01
Age groups, n (%)						
<44	110 (7.7)	1,566 (8.3)	0.19	100 (7.6)	104 (7.9)	0.03
45–64	581 (40.6)	6,532 (34.5)		529 (40.1)	529 (40.1)	
65–74	405 (28.3)	4,893 (25.9)		373 (28.3)	385 (29.2)	
75+	335 (23.4)	5,930 (31.3)		316 (24.0)	300 (22.8)	
CCI scores, mean ± SD	3.3 ± 2.5	3.1 ± 2.9	0.07	3.4 ± 2.6	3.7 ± 2.8	0.11
0	180 (12.6)	3,414 (18.0)	0.15	163 (12.4)	154 (11.7)	0.03
1	217 (15.2)	3,119 (16.5)		188 (14.3)	196 (14.9)	
2+	1,034(72.3)	12,388 (65.5)		967 (73.4)	968 (73.4)	
Follow-up years, mean ± SD	3.9 ± 3.4	4.2 ± 3.8	0.08	4.1 ± 3.5	4.3 ± 3.4	0.05
Comorbidities, n (%)						
Diabetic mellitus	943 (65.9)	8,052 (42.6)	0.48	860 (65.3)	863 (65.5)	<0.01
Hypertension	1,170 (81.8)	13,042 (68.9)	0.30	1,086 (82.4)	1,104 (83.8)	0.04
Hyperlipidemia	531 (37.1)	2,811 (14.9)	0.52	476 (36.1)	515 (39.1)	0.06
Coronary artery disease	411 (28.7)	3,106 (16.4)	0.30	395 (30.0)	391 (29.7)	0.01
Peripheral artery disease	154 (10.8)	1,540 (8.1)	0.09	148 (11.2)	160 (12.1)	0.03
Atrial fibrillation	96 (6.7)	1,284 (6.8)	0.00	93 (7.1)	91 (6.9)	0.01
Liver cirrhosis	141 (9.9)	3,070 (16.2)	0.19	137 (10.4)	130 (9.9)	0.02
COPD	177 (12.4)	2,796 (14.8)	0.07	173 (13.1)	171 (13.0)	<0.01
HBV	24 (1.7)	354 (1.9)	0.01	23 (1.7)	19 (1.4)	0.02
HCV	73 (5.1)	1,137 (6.0)	0.04	72 (5.5)	82 (6.2)	0.03
Dementia	66 (4.6)	1,150 (6.1)	0.07	63 (4.8)	73 (5.5)	0.03
Medications, n (%)						
Aspirin/clopidogrel	880 (61.5)	7,684 (40.6)	0.43	822 (62.4)	832 (63.1)	0.02
ACEI/ARB	1,147 (80.2)	12,210 (64.5)	0.35	1,077 (81.7)	1,074 (81.5)	0.01
Beta-blocker	1,115 (77.9)	11,393 (60.2)	0.39	1,034 (78.5)	1,045 (79.3)	0.02
Calcium channel blocker	1,237 (86.4)	13,956 (73.8)	0.32	1,137 (86.3)	1,147 (87.0)	0.02
K-sparing diuretics	172 (12.0)	2,126 (11.2)	0.02	159 (12.1)	152 (11.5)	0.02
Thiazide	140 (9.8)	1,407 (7.4)	0.08	132 (10.0)	123 (9.3)	0.02
Loop diuretics	1,102 (77.0)	12,419 (65.6)	0.25	1,023 (77.6)	1,034 (78.5)	0.02
Glucose-lowering drugs	827 (57.8)	6,197 (32.8)	0.52	752 (57.1)	770 (58.4)	0.03
Insulin	902 (63.0)	9,284 (49.1)	0.28	841 (63.8)	845 (64.1)	0.01
Statin	1,271 (88.8)	2,640 (14.0)	2.26	1,158 (87.9)	1,157 (87.8)	<0.01
Ketosteril	243 (17.0)	2,168 (11.5)	0.16	214 (16.2)	237 (18.0)	0.05
Lab (baseline)						
eGFR, ml/min/1.732 (Saran et al. (2017)	10.1 ± 3.4	9.8 ± 3.7	0.08	10.1 ± 3.4	10.2 ± 3.6	0.03
Hemoglobin, g/dL	9.9 ± 1.6	9.8 ± 1.7	0.05	9.9 ± 1.6	9.9 ± 1.6	0.03
K	4.4 ± 0.7	4.3 ± 0.8	0.01	4.4 ± 0.7	4.4 ± 0.7	<0.01
P	4.7 ± 1.4	4.5 ± 1.3	0.16	4.7 ± 1.3	4.7 ± 1.3	0.01
Ca	8.7 ± 0.8	8.7 ± 0.8	0.10	8.7 ± 0.8	8.7 ± 0.8	0.03
Albumin	3.6 ± 0.6	3.6 ± 0.6	0.04	3.6 ± 0.6	3.6 ± 0.6	0.04
BUN	60.9 ± 27.5	58.1 ± 28.7	0.10	60.4 ± 27.3	60.1 ± 28.0	0.01
LDL	131.5 ± 30.6	99.8 ±25.7	0.12	130.9 ± 29.8	130.5 ± 29.5	0.02

ASMD, absolute value of standardized mean difference; DM, diabetic mellitus; COPD, chronic obstructive pulmonary disease; HBV, hepatitis B virus; HCV, hepatitis C virus.

### Effect of statin on infection risks in patients with advanced chronic kidney disease

Table 2 presents the risk of IRH, MACE, and mortality in statin users compared with statin non-users. The infection-related hospitalization (IRH) rate was 79.3 (71.0–87.5) per 1,000 person-years in statin users and 94.3 (85.3–103.2) per 1,000 person-years in statin non-users. The Cox proportional hazards model with competing risk analysis revealed statin users had lower risk of IRH with a hazard ratio (HR) [HR, 0.83; 95% confidence interval (CI) 0.74–0.93, *p* = 0.002] compared with statin non-users. Regarding different causes of IRH, statin users had lower risk of sepsis (HR, 0.73; 95% CI 0.61–0.89, *p* = 0.001) and urinary tract infection (HR, 0.81; 95% CI 0.66–1.00, *p* = 0.049) compared to statin non-users. A trend toward decreased risk though not reaching statistical significance was observed in pneumonia (HR, 0.87; 95% CI 0.72–1.04, *p* = 0.13) and catheter related infection (HR, 0.92; 95% CI 0.74–1.16, *p* = 0.489).

**TABLE 2:** Event numbers and hazard ratios of the primary outcomes between statin users and statin non-users.

Outcomes	Statin users			Statin non-users			Hazard ratio (95% CI)^b^	*p* value
	No. of events (%)		Incidence rate (95% CI)^a^	No. of events (%)		Incidence rate (95% CI)^a^		
Infection-related hospitalization (IRH)	355	(26.9)	79.3 (71.0–87.5)	429	(32.5)	94.3 (85.3–103.2)	0.83 (0.74–0.93)	0.002
Sepsis	120	(9.1)	23.0 (18.9–27.1)	164	(12.4)	30.7 (26.0–35.4)	0.73 (0.61–0.89)	0.001
Lung infection	127	(9.6)	24.5 (20.3–28.8)	149	(11.3)	27.8 (23.3–32.2)	0.87 (0.72–1.04)	0.130
Urinary tract infection	91	(6.9)	17.5 (13.9–21.1)	115	(8.7)	21.4 (17.5–25.3)	0.81 (0.66–1.00)	0.049
Soft tissue infection	74	(5.6)	14.1 (10.9–17.3)	77	(5.8)	14.1 (10.9–17.2)	1.07 (0.83–1.37)	0.612
Catheter related infection	84	(6.4)	16.2 (12.7–19.7)	88	(6.7)	16.5 (13.0–19.9)	0.92 (0.74–1.16)	0.489
Major adverse cardiac event	195	(14.8)	38.9 (33.4–44.4)	286	(21.7)	55.9 (49.5–62.4)	0.72 (0.62–0.83)	<0.001
Myocardial infarction	85	(6.5)	16.1 (12.7–19.5)	159	(12.1)	29.5 (24.9–34.1)	0.54 (0.44–0.67)	<0.001
Heart failure	46	(3.5)	8.6 (6.1–11.1)	55	(4.1)	9.9 (7.2–12.5)	0.89 (0.67–1.20)	0.455
Stroke	88	(6.7)	16.7 (13.2–20.2)	107	(8.1)	19.7 (16.0–23.4)	0.90 (0.72–1.11)	0.323
All-cause mortality	569	(43.1)	104.1 (95.6–112.7)	670	(50.8)	118.2 (109.3–127.2)	0.89 (0.76–1.03)	0.123
Infection-related mortality	174	(13.5)	10.8 (8.0–13.6)	282	(21.3)	16.6 (13.2–19.9)	0.59 (0.38–0.92)	0.019
Cardio-related mortality	351	(26.4)	21.4 (17.5–25.3)	387	(29.4)	22.8 (18.9–26.7)	0.84 (0.59–1.19)	0.326

a Event numbers per 1,000 person-years.

b The competing risk analysis was performed for the incidence of interested outcomes except mortality.

### Effect of statin on major adverse cardiovascular events in patients with advanced chronic kidney disease

The incidence rate of MACE was 38.9 (33.4–44.4) per 1,000 person-years in statin users and 55.9 (49.5–62.4) per 1,000 person-years in statin non-users. The Cox proportional hazards model with competing risk analysis revealed statin users had lower risk of MACE (HR, 0.72; 95% CI 0.62–0.83, *p* < 0.001) compared to statin non-users. Statin user had lower risk of non-fatal myocardial infarction (HR, 0.54; 95% CI 0.44–0.67, *p* < 0.001) and a trend toward decreased risk of hospitalization for heart failure (HR, 0.89; 95% CI 0.67–1.20, *p* = 0.455) and non-fatal stroke (HR, 0.90; 95% CI 0.72–1.11, *p* = 0.323) compared with statin non-users.

### Effect of statin on mortality in patients with advanced chronic kidney disease

**FIGURE 2:**
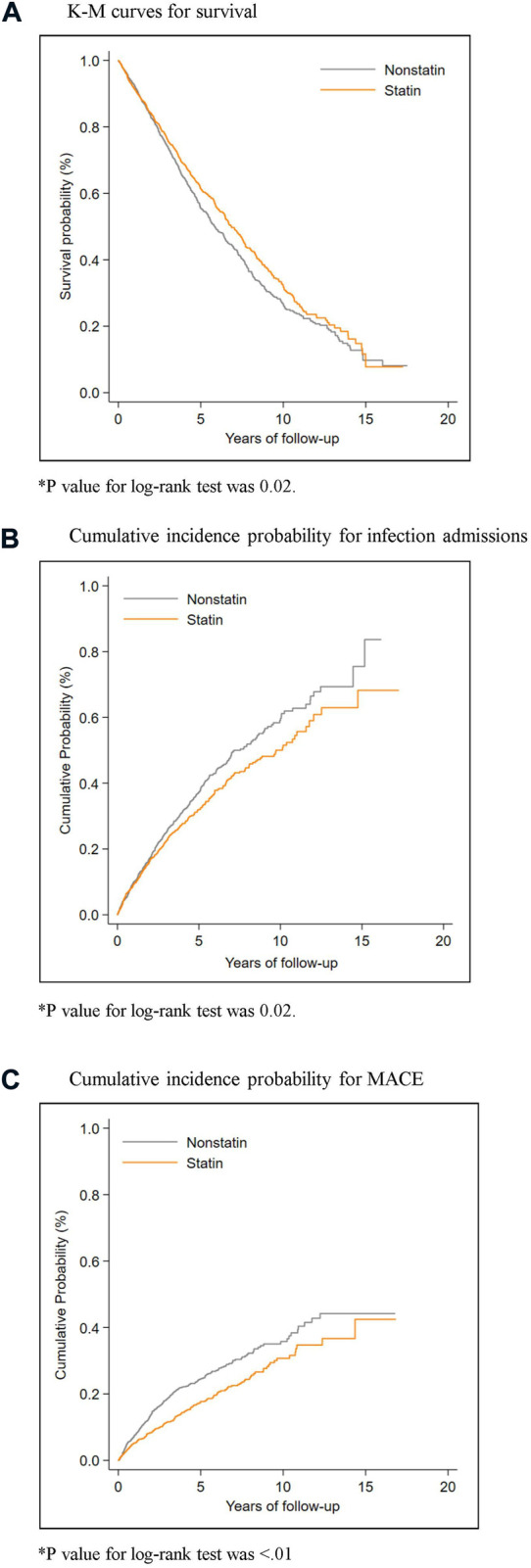
Cumulative probability curves for primary outcomes by statin exposure status.

The incidence rate of all-cause mortality was 104.1 (95.6–112.7) per 1,000 person-years in statin users and 118.2 (109.3–127.2) per 1,000 person-years in statin non-users. There was no significant mortality difference between statin users and statin non-users (HR, 0.89; 95% CI 0.76–1.03, *p* = 0.123). Regarding infection-related mortality, statin users had significant lower risk (HR, 0.59; 95% CI 0.38–0.92, *p* = 0.019) compared with statin non-users. Regarding cardiovascular related mortality, there was no significant difference between statin users and statin non-users [HR, 0.84; 95% CI 0.59–1.19), *p* = 0.326]. The cumulative survival curves for infection-related admissions, MACE, and mortality are shown in Figure 2.

### Subgroup analysis

**FIGURE 3:**
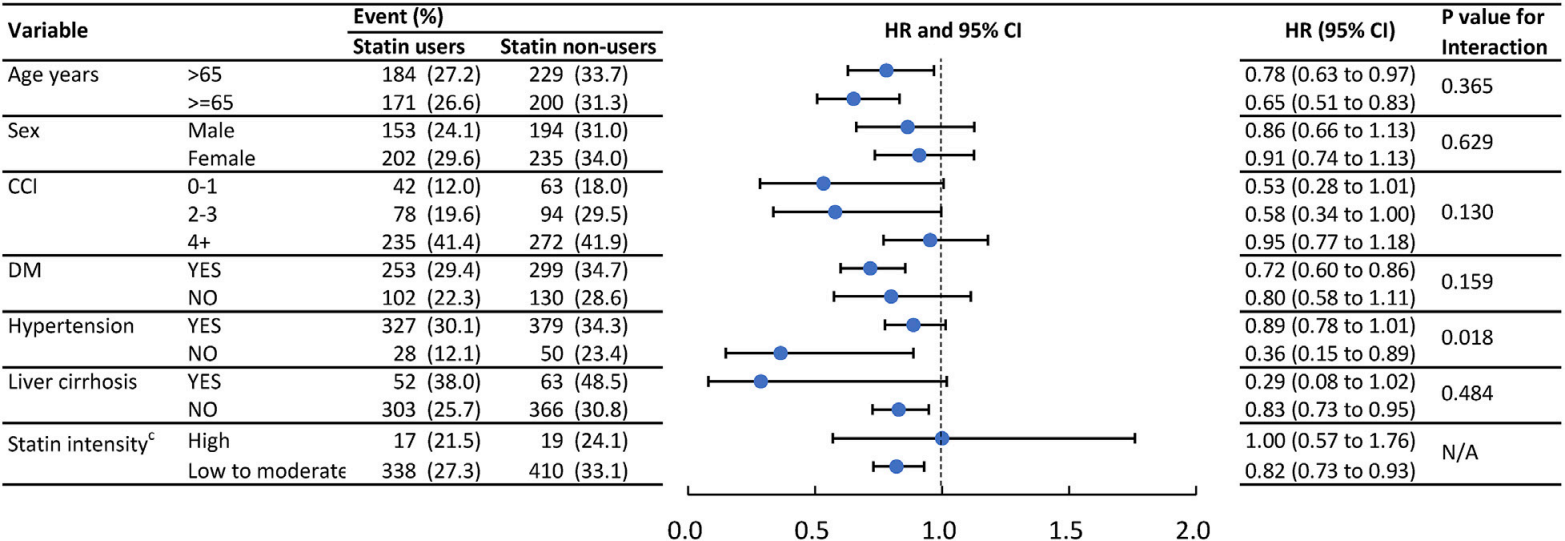
Event numbers and hazard ratios of the infection admissions between statin users and statin non- users.

**FIGURE 4:**
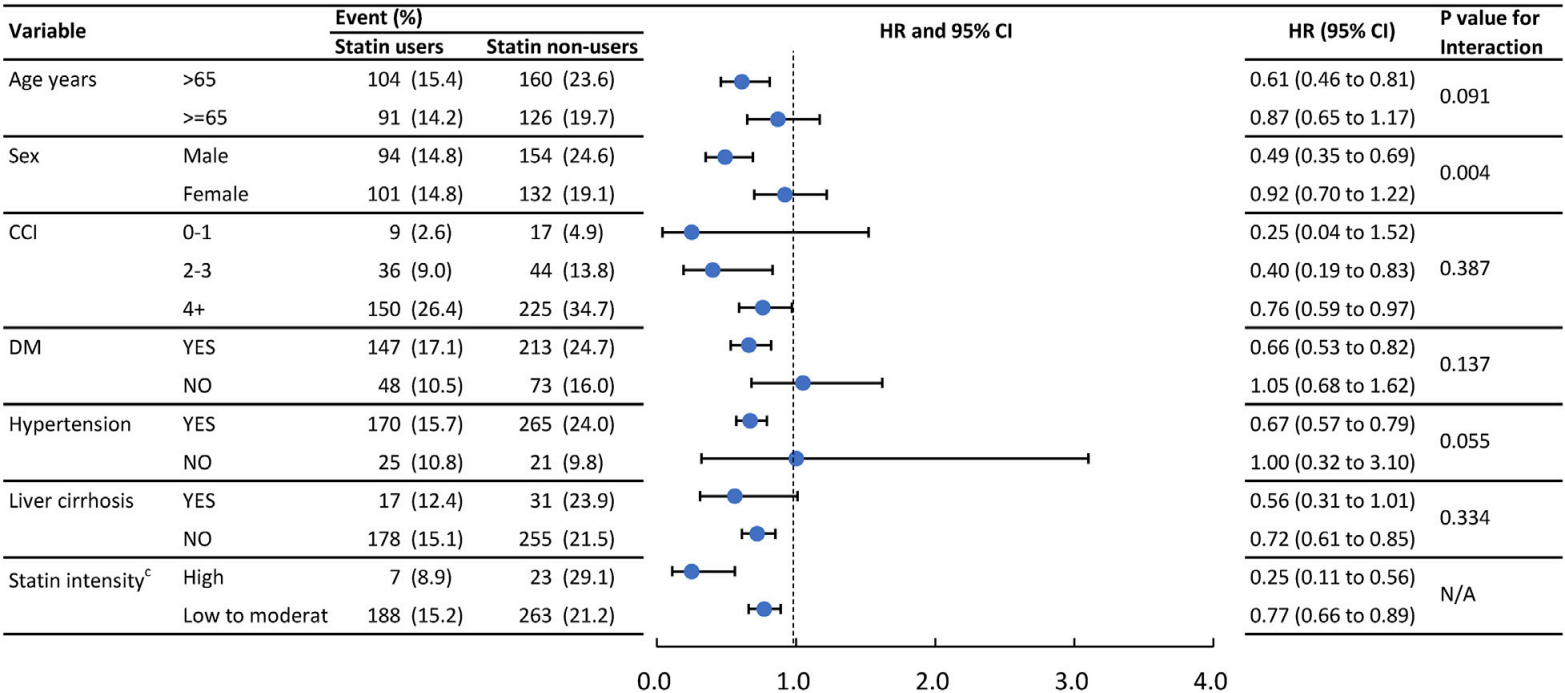
Event numbers and hazard ratios of the MACE between statin users and statin non-users.

Figure 3 presents the result of subgroup analysis of IRH and Figure 4 presents the result of subgroup analysis of MACE. To further verify consistent effects of statin among different clinical populations, we performed pre-specified subgroup analyses of age ≥55 or <55, gender, CCI score 0, 1, or 2+, CV comorbidity or not, with high-intensity statin or not, in outcomes of IRH and MACE. The *p* for interaction was not significantly different between groups except for hypertension in IRH and gender in MACE. Patients without hypertension benefit more from the protective effect of statin in IRH (*p* for interaction, 0.018). Male patients benefit more from the protective effect of statin in MACE (*p* for interaction, 0.004).

## Discussion

This is a multi-institutional observational study to evaluate the clinical effects of *de novo* statin use in patients with advanced CKD (stage V) before chronic dialysis. The key information is: 1) *de novo* statin use in patients with advanced CKD is associated with 17% lower risks of hospitalization for infection diseases (HR, 0.83; 95% CI 0.74–0.93, *p* = 0.002) and infection-related mortality (HR, 0.59; 95% CI 0.38–0.92, *p* = 0.019); 2) compared with statin non-users, patients with advanced CKD under statin treatment have lower-MACE rate during clinical follow-up (HR, 0.72; 95% CI 0.62–0.83, *p* < 0.001), mainly in reducing the event of myocardial infarction; and 3) nevertheless, statin treatment still does not extend survival duration in patients with advanced CKD. No difference of all-cause mortality was noted between statin users and statin non-users.

### Statin effects on infectious diseases and sepsis

Statins have been found to reduce CV events regardless of the baseline LDL cholesterol level and their protective effects are independently associated with the reduction of inflammatory factors, such as C-reactive protein (Ridker et al., 2005; Ridker et al., 2008). Several pleiotropic effects of statins, including normalized vasomotor response, anti-oxidant effects, immunomodulation properties, and stabilization of atherosclerotic plaques, are proposed. Anti-inflammatory and anti-bacterial properties in experimental studies indicated that statins may prevent development of sepsis, attenuate the severity of sepsis, and then improve survival in individuals at risk (Merx et al., 2004; Masadeh et al., 2012). Recently, Omar et al. reported that statin users had 12% lower risk of in-hospital death than statin non-users in patients admitted for severe coronavirus disease 2019 (COVID-19) (Saeed et al., 2020). A meta-analysis of 28 observational studies also concluded the use of statins among individuals with COVID-19 were significantly decreasing the need for invasive mechanical ventilator and all-cause mortality (Wu et al., 2021).

Patients with CKD are at high risk for sepsis and sepsis-related morbidity and mortality. Studies from observational cohorts demonstrated a graded risk for sepsis with decreasing estimated GFR levels (James et al., 2008). Patients with CKD stage 4 or 5 had 3.5-fold higher risk of sepsis than those with estimated GFRs > 60 ml/min/1.73 m^2^. For statin effects on sepsis among patients with advanced CKD, conducted a national prospective cohort study and reported the use of statins independently associated with a reduction of hospitalization for sepsis in 1,041 incident dialysis patients (Gupta et al., 2007). In a national-wide observational study, uses of simvastatin or atorvastatin were associated with 28% or 22% lower 30-day mortality in patients with sepsis (Lee et al., 2018). Our results demonstrated a consistent result that initiating statin in patients with CKD stage V reduced 17% risks of hospitalization for infection diseases. Furthermore, death from infection diseases was significantly less in statin users than statin non-users in our study cohort. Nevertheless, other studies have shown conflicting results regarding the effect of prior statin therapy on hospitalized patients with pneumonia or sepsis in general population (van den Hoek et al., 2011; Wan et al., 2014; Ghayda et al., 2021). Different study design, patient population, definitions of infectious diseases, statistical methods, and interest outcomes among these observational studies make controversial results. There is no doubt that the benefits of statin on outcomes of sepsis or specific infectious diseases should be justified and validated in randomized controlled trials. In our study cohort, there were vulnerable CKD stage V patients with very high risks for sepsis or infectious diseases, compared with general or heterogenous population in other observational studies. Statin beneficial effects on sepsis prevention and survival improvement may tend to be disclosed among patients at the heightened risk for sepsis and sepsis-related mortality. This hypothesis should be confirmed in a further large scaled cohort study analyzing outcomes of sepsis in patients with risk stratification.

### Statin treatment in chronic kidney disease

Patients with CKD have a substantially increased risk of CVD. Risk of coronary events in patients with CKD stage 3 or 4 is equivalent or higher than those with diabetes (Tonelli et al., 2012). Statin has been proven to reduce the risk of CV mortality and atherosclerotic events, even among patients with CKD not undergoing dialysis (Baigent et al., 2011b; Upadhyay et al., 2012; Palmer et al., 2014). Current concepts for lipid management suggest starting statin treatment depends on the individual risk of atherosclerotic CVD, rather than a specific low-density lipoprotein (LDL) cholesterol level. Therefore, the Kidney Disease Improving Global Outcomes (KDIGO) foundation recommends that universal statin treatment for individuals ≥50 years of age with CKD (Wanner et al., 2014). The American College of Cardiology/American Heart Association (ACC/AHA) guidelines do not specifically address CKD and endorses statins for adults with atherosclerotic CVD, LDL cholesterol ≥190 mg/dl, diabetes, or a 10-year predicted risk for atherosclerotic CVD ≥ 7.5% (Goff et al., 2014). Even through different criteria for statin use, these guidelines have high concordance for identifying adults indicated for appropriate use of statin. Although patients with CKD are known as the highest risk group for CV events, these patients are usually undertreated and only half of them are taking statins (Colantonio et al., 2015). Physicians did not prescribe statin in this population probably because of side effects, polypharmacy, multiple comorbidities, lack of awareness, and uncertain benefit in advanced diseases. Randomized control trials including 4D (Die Deutsche Diabetes Dialysis), AURORA (A Study to Evaluate the Use of Rosuvastatin in Subjects on Regular Hemodialysis) (Wanner et al., 2005b), and subgroup analysis of SHARP (Study of Heart and Renal Protection) (Baigent et al., 2011b) all reported statins did not reduced events of CVD death, myocardial infarction, or stroke in patients with chronic hemodialysis. Generally, the presence of competing risks for sudden death or non-CV events explains the reasons for lack of statin benefit in patients with chronic hemodialysis. These results may not conclude statin effects in preventing or halting the progression of atherosclerosis that do not exist in this population.

Patients with CKD stage V have less or similar status of cardiovascular morbidities and competing risks with chronic hemodialysis. Limited evidence is available for benefits of statin use in this specific population. We focused on patients with CKD stage V in our study. Our results demonstrated initiating statins in patients with CKD stage V that reduced 28% risk of MACE than those without statin use. The difference was mainly from the rate of myocardial infarction, which assure statin effects on atherosclerotic CVD prevention in advanced CKD. Nevertheless, all-cause mortality was not different between statin users and statin non-users in the cohort. Competing risks for death from fatal complications of renal failure and cardiovascular morbidity, such as electrolyte imbalance or life-threatening cardiac arrhythmias may omit the survival benefit of statin in patients with CKD stage V. We did not interpret these results as lacking benefit of statin, but more comprehensive and multidiscipline care was needed to settle with multiple complex medical issues for survival improvement in these patients. Furthermore, some patients with advanced CKD confronted with a difficult decision-making of life with chronic dialysis or passive palliative care because of economic issues, supporting systems, or personal perspective issues. Individuals with palliative treatment also attenuated the differences between statin users or statin non-users.

Our results added a piece of puzzle in clinical evidence of statin treatment in patients with CKD. Statin use is never too late in patients with CKD stage V in term of atherosclerotic CVD prevention. Increasing appropriate use of statins in CKD patients should be emphasized and implement further in clinical practice.

Our study has certain limitations. This was a retrospective and observational cohort study. First, the observational studies of specific drugs are limited to confounding by indication for treatment. Statins are commonly used in primary or secondary prevention of atherosclerotic diseases. People who take statins have engaged in the systemic health care and acquired a comprehensive disease treatment and surveillance plan. Otherwise, people who do not take statins may not afford cost of medical care or not behave a healthy lifestyle to prevent disease development. To deal with this important confounder, we included patients who are subjected to tract longitudinally in programmed CKD care and had at least 1 year health care records into the study analyses. Patients with documented atherosclerotic CVD before index date were excluded. We compared the primary or secondary outcomes in a propensity-matched cohort who had similar likelihoods of being prescribed a statin to control many potential confounders. Second, though CGRD is the largest multi-institutional electronic medical records database in Taiwan, identifying subjects receiving statin treatment or presenting the interested outcome in other hospitals is challenging, which may bias the results. Third, to evaluate the statin effects in particular population (CKD stage V), we defined statin exposure as patients had the prescription of a certain stain within 90 days of the date of a diagnosis of CKD stage V. Patients with prior statin use in earlier CKD course were excluded. We did not deal with the confounder of alternations of drug dosage or regimen during the study period. We did not have complete prescription and refill information for analyses because patients are not obliged to take medications in our health care facilities. The information of drug compliance in the study, which patients who are prescribed and take them regularly, could not be obtained. After controlling for confounding through aforementioned methods, we find a statistically association between statin use and the risk of death or hospitalization for infection diseases. Furthermore, we provided clinical evidence of the MACE reduction of statin use in patients with CKD V. Nevertheless, the unmeasured confounders still exist and might affect our results. A further large scaled prospective or randomized trials may need to confirm our results.

## Conclusion

Anti-inflammatory properties of statin are observed in experiments and many clinical trials. Studies on statin effects of prevention and survival improvement in sepsis or infectious diseases in general population yielded conflicting results. Patients with CKD have increased risk for sepsis, with graded risk as estimated GFR decreasing. Our study showed initiating statins in patients with CKD stage V, one of the most susceptible groups, reduce rates of hospitalization and mortality for sepsis and infectious diseases. Further research or clinical trials are needed to confirm our observations and hypothesis.

For patients with impair or moderate renal impairment, the role of statins in prevention of CVD has been confirmed in meta-analyses or randomized trials. Nonetheless, parallel benefits of statins did not show among individuals with dialysis-dependent CKD. A doubt of cost-effectiveness of statins in individuals with advanced CKD before chronic dialysis is aroused. Our study provided valuable evidence of benefits of statins on MACE reduction, particularly on myocardial infarction, in CKD stage V patients. Apart from dialysis dependent CKD, the statin use should be based on individual risk of atherosclerotic CKD, regardless of severity of renal impairment. Underuse of statins is common in CKD population and how to overcome the barrier to widespread application in statin beneficial individuals will be the next crucial task.

## Data Availability

The raw data supporting the conclusion of this article will be made available by the authors, without undue reservation.
