# Supplementary material for: Statin uses in adults with non-dialysis advanced chronic kidney disease: Focus on clinical outcomes of infectious and cardiovascular diseases
Source: Front Pharmacol. 2022 Sep 30;13:996237. doi: 10.3389/fphar.2022.996237 (PMC9561676; doi:10.3389/fphar.2022.996237)
Supplement: Supplementary file 1 [file DataSheet1.docx]

**Supplementary Content**

Table 1. ICD-9 and ICD-10 Definitions for Covariates and Outcomes.

| **Covariate** | **ICD-9 & ICD-10 codes** |
| --- | --- |
| Diabetic mellitus | 250.x, E10.x -E14.x |
| Hypertension | 401.x, 402.x, I10.x -I15.x |
| Hyperlipidemia | 272.x, E78.5 |
| Coronary artery disease | 411.x, 413.x, 414.01, 414.02, 414.03, 414.04, 414.05, 414.8, 414.9, I25.0, I25.1, I25.3, I25.4, I25.5, I25.6, I25.7, I25.8, I25.9 |
| Peripheral artery disease | 440.0-443.9, 447.1, 785.4, I70.x, I71.x, I73.1, I73.8, I73.9, I77.1, I79.0, I79.2, K55.1, K55.8, K55.9, Z95.8, Z95.9, 38.13-38.14(P)*, 38.16(P)*, 38.18(P)*, 38.33-38.34(P)*, 38.36(P)*, 38.38(P)*, 38.43-38.44(P)*, 38.46(P)*, 38.48(P)*, 38.22-38.26(P)*, 39.29(P)* |
| Atrial fibrillation | 427.31, I48.x |
| Liver cirrhosis | 571.2, 571.5, 571.6, 571.8, 571.9, B18.x, K70.0, K70.1, K70.2, K70.3, K70.9, K71.3, K71.4, K71.5, K71.7, K73, K74, K76.0, K76.2, K76.3, K76.4, K76.8, K76.9, Z94.4 |
| Chronic Obstructive Pulmonary Disease, COPD | 415.0, 416.8-416.9, 491-494, 496.x, I27.8, I27.9, J40.x–J47.x, J60.x–J67.x, J68.4, J70.1, J70.3 |
| HBV | 702.x, 703.x, B18.0, B18.1 |
| HCV | 704.1, 704.4, 705.1, 705.4, 707.x, B18.2 |
| Dementia | 290.x, 331.x, 331.0, 331.1, 331.2, F00.x, F01.x, F02.x, F03.x, F05.1, G30.x, G31.1 |
| **Outcome** |  |
| Infection related hospitalization | 038.x, 995.91, 995.92, 020.2, 785.52, 790.7, R65.20, R65.21, R78.81, A41.9, A49.9, B96.89, 481.x, 482.x, 483.x, 485.x, 486.x, 510.0, 510.9, J14.x, J15.x, J16.x, J17.x, J18.x, J12.x, J69.x, J95.8, J09.x, M96.x, B25.0, B96.1, B95.3, B37.1, B38.0, A74.x, A40.3, A02.22 B45.0, B44.9, O89.01, J85, J86, 590.x, 595.0, 599.0, N39.0, N30.01, N30.90, N99.51, B37.41, A54.01, A56.01, N10.x, N11.x, N12.x, N15.x, N16.x, T83.511, 681.x, 682.x, 728.86, 711.x, 730.3, 730.8, 730.9, L03.x, H60.1, H05.01, H00.03, K61.x, M72.6, M00, M19.90, M86, M46.2, M46.3, M46.4, M46.5, 996.6, 999.3, T80.2, T82.7, T85.7, T83.51, |
| Sepsis | 038.x, 995.91, 995.92, 020.2, 785.52, 790.7, R65.20, R65.21, R78.81, A41.9, A49.9, B96.89 |
| Lung infection | 481.x, 482.x, 483.x, 485.x, 486.x, 510.0, 510.9, J14.x, J15.x, J16.x, J17.x, J18.x, J12.x, J69.x, J95.8, J09.x, M96.x, B25.0, B96.1, B95.3, B37.1, B38.0, A74.x, A40.3, A02.22 B45.0, B44.9, O89.01, J85, J86 |
| Urinary tract infection | 590.x, 595.0, 599.0, N39.0, N30.01, N30.90, N99.51, B37.41, A54.01, A56.01, N10.x, N11.x, N12.x, N15.x, N16.x, T83.511 |
| Soft tissue infection | 681.x, 682.x, 728.86, 711.x, 730.3, 730.8, 730.9, L03.x, H60.1, H05.01, H00.03, K61.x, M72.6, M00, M19.90, M86, M46.2, M46.3, M46.4, M46.5 |
| Catheter related infection | 996.6, 999.3, T80.2, T82.7, T85.7, T83.51 |
| Myocardial infarction | 410.x, I21.x, I22.x |
| Heart failure | 428.x, I50.x |
| Stroke | 430.x, 431.x, 432.x, 433.x, 434.x, 435.x, 436.x, 437.x, I60.x, I61.x, I62.x, I63.x, I64.x, G45.0, G45.1, G45.4, G45.8, G45.8, I67.x |

*Mean ICD-9 procedure codes

Figure 1. Histograms Showing the Density of Propensity Score Distribution in Statin User and Statin Non-User Groups before and after Matching.

| 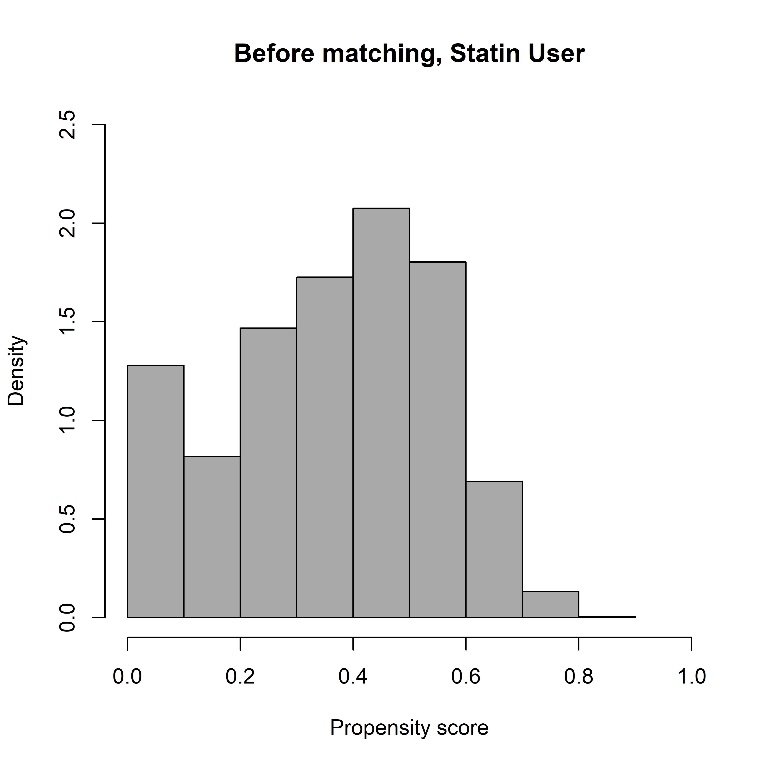 | 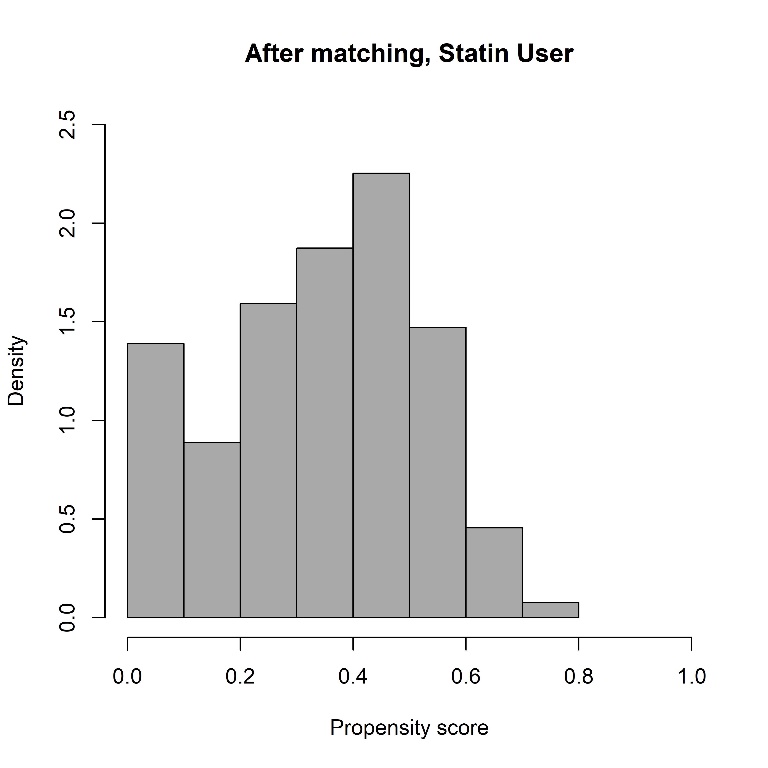 |
| --- | --- |
| 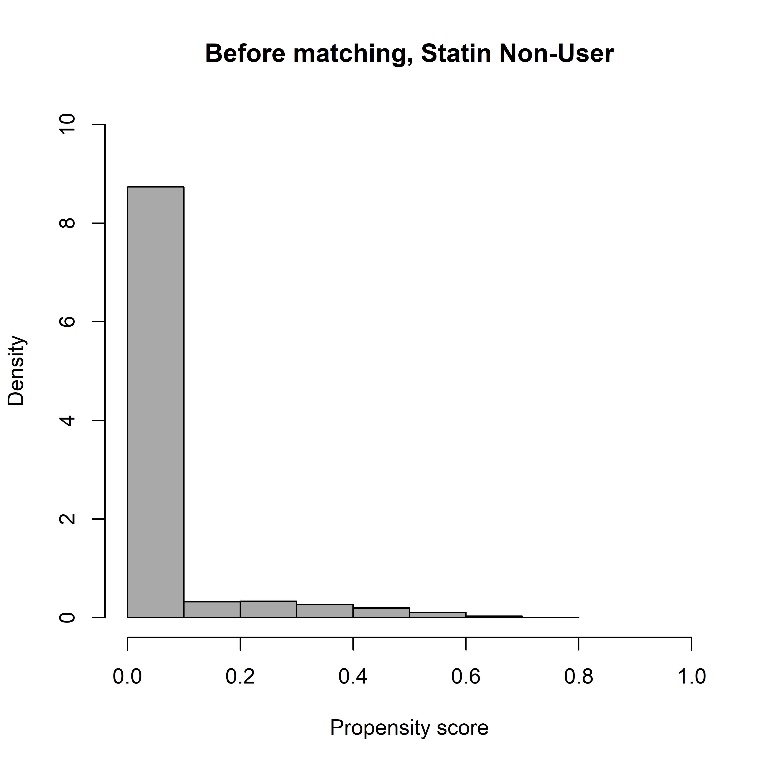 | 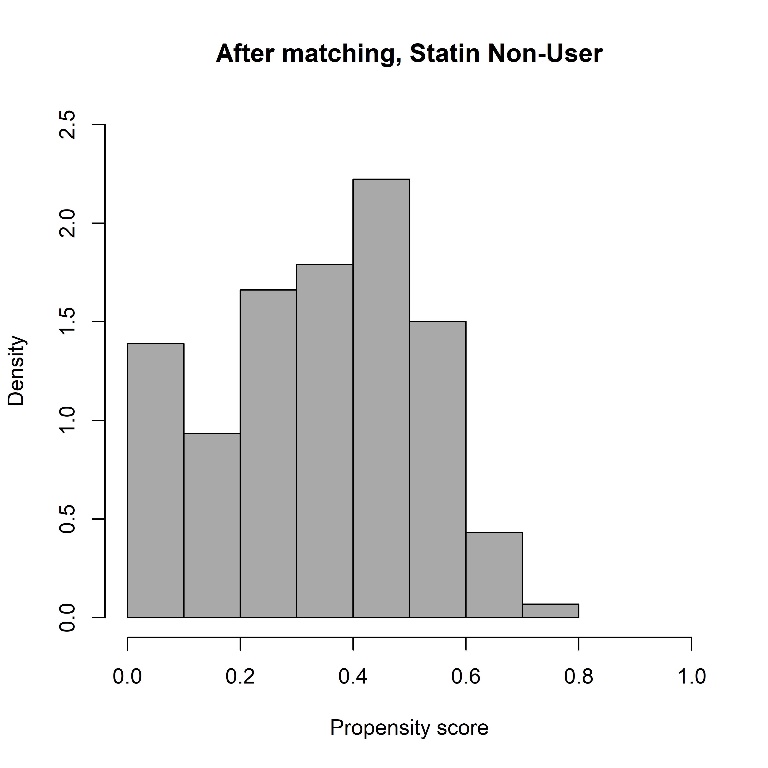 |
